# A Comprehensive Analysis of 21 Actionable Pharmacogenes in the Spanish Population: From Genetic Characterisation to Clinical Impact

**DOI:** 10.3390/pharmaceutics15041286

**Published:** 2023-04-19

**Authors:** Rocio Nunez-Torres, Guillermo Pita, María Peña-Chilet, Daniel López-López, Jorge Zamora, Gema Roldán, Belén Herráez, Nuria Álvarez, María Rosario Alonso, Joaquín Dopazo, Anna Gonzalez-Neira

**Affiliations:** 1 Human Genotyping Unit (CEGEN), Cancer Genetics Program, National Cancer Research Center (CNIO), 28029 Madrid, Spain; 2 Computational Medicine Platform, Fundación Progreso y Salud (FPS), Hospital Virgen del Rocío, 41013 Sevilla, Spain; 3 Bioinformatics in Rare Diseases (BiER), Centre for Biomedical Network Research on Rare Diseases (CIBERER), ISCIII, 41013 Sevilla, Spain; 4 Computational Systems Medicine Group, Institute of Biomedicine of Seville, IBiS, University Hospital Virgen del Rocío/CSIC/University of Sevilla, 41013 Seville, Spain; 5 Functional Genomics Node, FPS/ELIXIR-ES, Hospital Virgen del Rocío, 41013 Sevilla, Spain; 6 Centre for Biomedical Network Research on Rare Diseases (CIBERER-U706), ISCIII, 28029 Madrid, Spain

**Keywords:** pharmacogenetics, pharmacogenomics, Spanish population, pharmacogenetics characterisation, genotyping, sequencing, population, database

## Abstract

The implementation of pharmacogenetics (PGx) is a main milestones of precision medicine nowadays in order to achieve safer and more effective therapies. Nevertheless, the implementation of PGx diagnostics is extremely slow and unequal worldwide, in part due to a lack of ethnic PGx information. We analysed genetic data from 3006 Spanish individuals obtained by different high-throughput (HT) techniques. Allele frequencies were determined in our population for the main 21 actionable PGx genes associated with therapeutical changes. We found that 98% of the Spanish population harbours at least one allele associated with a therapeutical change and, thus, there would be a need for a therapeutical change in a mean of 3.31 of the 64 associated drugs. We also identified 326 putative deleterious variants that were not previously related with PGx in 18 out of the 21 main PGx genes evaluated and a total of 7122 putative deleterious variants for the 1045 PGx genes described. Additionally, we performed a comparison of the main HT diagnostic techniques, revealing that after whole genome sequencing, genotyping with the PGx HT array is the most suitable solution for PGx diagnostics. Finally, all this information was integrated in the Collaborative Spanish Variant Server to be available to and updated by the scientific community.

## 1. Introduction

The implementation of precision medicine is one of the main goals in medicine nowadays. One of the main milestones of precision medicine is pharmacogenetics (PGx), which is the study of a patient’s genomic data to achieve safer and more effective therapies [1]. In this sense, the implementation of PGx in clinical practice aims to reduce the impact of adverse drug reactions (ADRs). ADRs are a serious clinical problem, with 2.2 million ADRs annually (100,000 of which lead to fatal consequences), and they have a direct medical cost of 200 billion US dollars in the United States (US) [2]. Although big PGx initiatives have demonstrated PGx’s utility in the clinical setting [3,4,5,6] and the cost-effectiveness of PGx testing has also been established [7], mostly, PGx diagnostic is implemented in a reactive way, testing only those genes related with the drug which is going to be prescribed. However, pre-emptive PGx testing by a single test covering a sufficient number of medications and future medication exposure may be easier to implement and more cost-effective than reactive PGx testing.

In order to facilitate and promote the implementation of pre-emptive PGx testing in clinical practice, international initiatives have been developed. One of the most useful initiatives has been the creation of reference drug-gene interaction clinical guidelines, mainly through the Clinical Pharmacogenetics Implementation Consortium (CPIC) [8] of the US and the Dutch Pharmacogenetics Working Group (DPWG) of Europe [9], as well as the PGx database, PharmGKB [10], which offers a categorisation of the drugs and genes according to their evidence level from 1A to 4. Additionally, large-scale PGx characterisation using data from the UK Biobank initiative [11] has provided information about the main PGx characteristics of 14 pharmacogenes (PGx genes). Nevertheless, although the UK Biobank initiative is a powerful tool, population differences in the PGx variants have been reported [12,13,14] and disproportionate rates of uncatalogued deleterious variants have been shown, highlighting the need for large-scale PGx studies of diverse populations [15,16,17,18,19]. In this sense, the Spanish population is very homogeneous between the different Spanish regions, and it has been described as similar to other European populations but more genetically diverse than Western and Northern Europeans [20]. This fact is mainly due to the fraction of North-West African ancestry (0–11%) in Spanish population, related to an admixture event occurring during the Muslim rule in Iberia [21]. Of note, we have already observed these differences in the Spanish population [22].

To facilitate PGx implementation in the Spanish population, we performed a comprehensive PGx characterisation of more than 3000 individuals from Spain to address the following objectives: (i) to establish the reference frequencies of all the PGx genes included in CPIC guidelines (21 PGx genes) to be used as reference for the Spanish population; (ii) to determine putative pathogenic variants in order to create a catalogue of variants with a potential impact on PGx; (iii) to compare the main PGx diagnostic techniques; and (iv) to provide all the PGx information in a public database that is available to the scientific/clinical community.

## 2. Materials and Methods

### 2.1. Subjects and Genotyping

A total of 3006 Spanish individuals were used in the present study. The genetic data of 2046 individuals from the Collaborative Spanish Variant Server (CSVS) [22] were used in this project (Figure S1). All of them were Spanish individuals from different locations across the country and exomic or genomic aggregated data were available [22]. This server contains genetic information from unrelated samples of Spanish origin from the Medical Genome Project (EGA, accession: EGAS00001000938), other healthy controls, patients of different diseases, accompanied, in some cases, by unrelated phenotypically healthy carriers [22]. The sequences were contributed by more than 30 different consortiums and projects (see http://csvs.babelomics.org/ (accessed on 15 October 2021) for detailed information of all projects which contribute to this initiative).

In addition, 960 DNA samples from Spanish individuals provided by the Spanish National Biobank (BNADN) were also included in the study (Figure S1). These DNA samples belong to the Spanish population control collection from BNADN, which is a sample collection from healthy donors born in Spain (not diagnosed with any relevant disease) collected with the collaboration of the Regional Blood Transfusion Centres and blood banks. The samples were representative of the Spanish population since they were equally distributed between sex (480 females and 480 males) and were collected across the country, including covering the different regions and the possible genetic variability of Spain.

DNA samples from BNADN were genotyped using a standard high-throughput (HT) array, the Illumina Global Screening Array v1.0+MD, containing 700,078 SNVs (including more than 100,000 SNPs related with clinical research), according to the manufacturer’s protocols, and they were scanned in an iScan system. GenomeStudio software v2.0.4 (Illumina) was used for genotype calling and PLINK [23] software for genotype extraction.

Additionally, in order to establish a benchmark of different PGx diagnostic techniques, 102 iberian (IBS) individuals included in the 1K Genomes Project [24] were analysed. DNA specimens were provided by the Spanish National Biobank (BNADN). Genotyping was performed using a standard HT array, Global Screening Array v1.0+MD (Illumina), and a PGx array, Infinium Global Diversity Array with Enhanced PGx (Illumina), which contains 1,954,642 SNVs (including 11,705 PGx variants from 1836 genes) according to the manufacturer’s protocols. GenomeStudio software v2.0.4 (Illumina) was used for genotype calling and PLINK [23] software for genotype extraction.

### 2.2. Pharmacogenetic Gene Selection

Those genes included in the CPIC guidelines and those genes with germline variants categorised as level 1A in the PharmGKB database (last access: 16 August 2021) were considered as main PGx genes for PGx population characterisation. A total of 21 PGx genes (*CACNA1S*, *CFTR*, *CYP2B6*, *CYP2C19*, *CYP2C9*, *CYP2D6*, *CYP3A5*, *CYP4F2*, *DPYD*, *F5*, *G6PD*, *HLA-A*, *HLA-B*, *IFNL3*, *MT-RNR1*, *NUDT15*, *RYR1*, *SLCO1B1*, *TPMT*, *UGT1A1* and *VKORC1*) were included in this study (Table S1). The *EGFR* gene was not included in this study since, although it is associated with a variant categorised as PharmGKB 1A level, this variant corresponds to a somatic variation. Additionally, some PGx variants categorised as 1A level by PharmGKB located in the surroundings of the predefined genes were also included (Figure S1).

For discovery PGx analysis, all the genes reported in the clinical annotations from the PharmGKB database [25] (last access: 16 August 2021) were used, with a total of 1045 genes being included (Table S2).

### 2.3. Pharmacogenetic Data Interpretation

The genetic data from whole-exome sequencing (WES), whole genome sequencing (WGS) and array genotyping were extracted in vcf format for each individual. In order to obtain PGx haplotype, PGx phenotype and clinical recommendations, PharmCat v.1.3. software was used [26]. The *G6PD* gene was interpreted with Stargazer v.1.0.8. software [27] and *CYP2D6* with Cyrius [28] software, and the PGx diplotype was subsequently integrated in the PharmCat tool in order to obtain the phenotypes and clinical recommendations for both genes.

The PGx genes not included in the previously mentioned software (*F5* and *MT-RNR1*) were interpreted using a custom Python script which obtains PGx level 1A variant genotype from vcf files and performs the interpretation, assigning the corresponding allele, phenotype and clinical recommendation in the case of the presence of the variant. In the case of *HLA-A* and *HLA-B* gene allele designation, we used Genotype Imputation HLA (Minimac4) 1.5.8 [29] from Michigan Imputation server to obtain a two-field (four-digit) allele. Information about the allele, phenotype and clinical recommendations were collected from the corresponding CPIC guidelines [8]. All the data from genotypes, phenotypes and recommendations were collected and plotted using ggplot [30] from R environment [31].

### 2.4. Allele Nomenclature Considerations

Data from CSVS belongs to a multicentric collaborative project where the different centres have had their data included in vcf format [22]. For the establishment of *alleles, phenotypes and PGx recommendation, only phased data from CSVS, corresponding to 1070 out of 2046 individuals, were used for the correct assignment of *alleles. In addition, genomic positions without variation in our population (monomorphic variants) were not provided in the files. Variants covered by WES or WGS with a minor allele frequency (MAF) < 10^−3^ were considered as monomorphic variants for calling purposes.

Allele interpretation was performed with the genetic information available for each individual. Since different techniques were used, the coverage of the PGx variants differ. In this case, the proportions provided are based only on those individuals where all of the main PGx functional variants of each PGx gene (associated with a function alteration in PharmVar [32] or included in the CPIC guidelines [8]) are covered. As a result, the number of individuals used for the characterisation of each gene differ according to the coverage of the gene by each technique.

Finally, when all positions are not available, PharmCAT and Stargazer software are not able to differentiate between the different *alleles offering different alternatives. In these cases, we considered the most frequent allele in accordance with CPIC frequency tables [8] as the most probable *allele; however, we cannot rule out the other alleles. Usually, all these different genotypes shared the same functional variant and, therefore, they shared the same phenotype and clinical recommendation. In the event that different phenotypes were predicted, an undetermined phenotype was assigned.

### 2.5. Allele Frequency Comparison

Frequencies of each genotype were calculated and compared with global frequencies in the Caucasian/European population reported in CPIC for those genes [8]. When genotype frequencies were not reported in CPIC, they were calculated using the Hardy-Weimberg equilibrium principle from the allelic frequencies reported. Differences between populations were estimated using an exact conditional test.

### 2.6. Putative Deleterious Variants

For putative deleterious variant analysis, the 2046 individuals from the CSVS cohort were used. Firstly, putative deleterious variants of main PGx genes were explored in order to identify putative high clinical impact variants. For this purpose, those variants detected in the 21 PGx genes included in this study and the surrounding ones (5 kb upstream and downstream) (Table S1), which are not present in PharmGKB database, were evaluated. Secondly, with the objective of creating a catalogue of putative deleterious variants related with PGx in the Spanish population, those variants identified in any of the genes from the PharmGKB database (1045 genes, Table S2) were evaluated. Considered as putative deleterious were variants defined according to the Variant Effect Predictor [33] as those having the following consequence types in their PGx gene: frameshift, splice acceptor, splice donor, start lost, stop gained, stop lost, transcript ablation and transcript amplification. Additionally, missense variants were included as putative missense deleterious variants if they were categorised as deleterious by two of the following predictors: SIFT [34] (>0.05), Polyphen [35] (<0.91), CADD [36] (>15) or GERP [37] (>2). Those variants not observed in GnomAD [38] were considered as novel and possibly private in the Spanish population. The variants were named according to standard nomenclature recommendations stated for genetic studies [39].

### 2.7. Comparison of Different Pharmacogenetic Diagnostic Techniques

For comparative purposes, the following techniques were included: (i) genotyping with a standard HT array, Global Screening Array v1.0+MD (Illumina) containing 700,078 SNVs (including more than 100,000 SNVs related with clinical research); (ii) genotyping with a PGX array, Infinium Global Diversity Array with Enhanced PGx (Illumina), which contains 1,954,642 SNVs (including 11,705 PGx variants from 1836 genes); and (iii) WES using Nextera (Illumina) + IDT exome research panel (IDT), spanning 39 Mb of the human genome covering 19,396 genes; (iv) WGS using Illumina technology. For each technique, the coverage of the genomic positions of the 214 level 1A variants reported in the PharmGKB database (last access: 16 August 2021) was obtained and data were presented as a percentage of coverage for each of the 21 genes evaluated.

### 2.8. Collaborative Spanish Variant Server (CSVS) Integration

The CSVS is a crowdsourcing initiative to provide information about the genomic variability of the Spanish population to the scientific/medical community [22]. PGx data were obtained from the PharmaGKB data portal [40]. These data were integrated in the CSVS server so that all of the genes contained in the PharmGKB database appear with links to all their associated PGX information, such as PharmaGKB level, drug, phenotype or CPIC guideline if available. In addition, frequencies of polymorphisms corresponding to PGx alleles identified in our population were also provided.

## 3. Results

### 3.1. Pharmacogenetic Genotypes and Phenotypes

**Figure 1:**
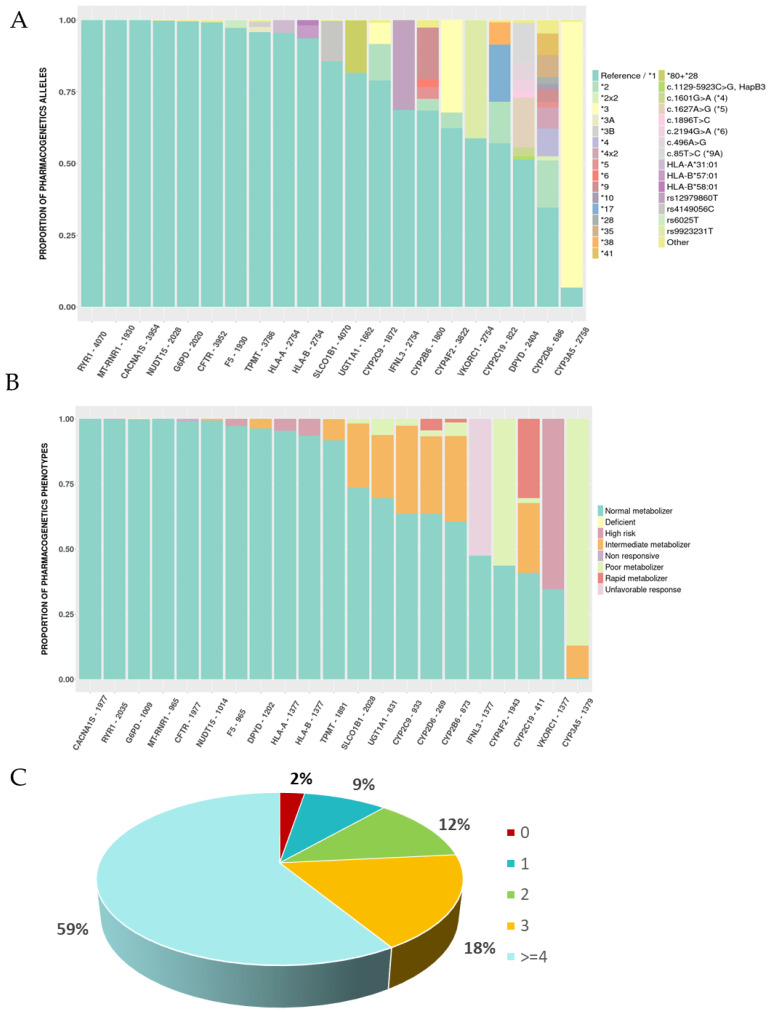
Pharmacogenetic genotype and phenotype proportions of the Spanish population for the main 21 pharmacogenetic (PGx) genes. (**A**) Proportion of the different PGx alleles (star (*) alleles) or variants for each of the 21 genes with the highest PGx levels of evidence (1A) and associated with the CPIC clinical guidelines identified in the Spanish population. Those alleles identified in <1% of the population were classified in the ‘other’ category. No-call genotypes have been removed for visual purposes, but a full list of the alleles identified is provided in Tables S3 and S4. (**B**) Proportion of the different pharmacogenetic phenotypes for each of the 21 genes with the highest PGx level of evidence (1A) and associated with the CPIC clinical guidelines identified in the Spanish population. No-call phenotypes have been removed for visual purposes, but a full list of the phenotypes identified is provided in Table S5. (**C**) Distribution of the number of actionable PGx variants associated with a therapeutical change in accordance with a clinical guideline in the Spanish population, in accordance with the number of actionable alleles they harbour.

PGx allelic characterisation of the Spanish population for the main 21 actionable pharmacogenes, those associated with a therapeutical change, is depicted in Figure 1A (Tables S3 and S4). No variability was detected in the Spanish population in *CACNA1S*, *MT-RNR1* and *RYR1*, whereas very low variability (AF < 7%) was described for *CFTR*, *F5*, *G6PD*, *HLA-A*, *HLA-B* and *NUDT15* (Figure 1A). On the contrary, the most variability was found in *CYP3A5*, with 93.2% of the alleles detected being the non-reference allele, followed by *CYP2D6* (65.3%), *DPYD* (48.6%), *CYP2C19* (42.9%), *VKORC1* (41.2%), *CYP4F2* (37.7%), *CYP2B6* (31.5%), *IFNL3* (31.3%), *CYP2C9* (21%), *UGT1A1* (18.3%) and *SLCO1B1* (14.3%) (Figure 1A). We must note that copy number alterations (CNVs) of *CYP2D6* were identified in 19.18% of individuals, but a vast majority of the duplications found (77.3%) were associated with the non-functional allele *CYP2D6* *4, not altering the functionality of the genotype. The proportions found in this study correlate with the frequencies described for the European population previously [11,17]. In this sense, no big differences were found when allele frequencies were compared with those described by the CPIC for a Caucasian population, with only slight differences identified in some alleles of *CYP2B6*, *CYP2D6* and *HLA-A/B* (Table S3).

Phenotype information showed, as expected, a correlation with the genotype characterisation of the population (Figure 1B, Table S5). In this sense, those genes with no or low variability (*CACNA1S*, *MT-RNR1*, *RYR1*, *CFTR*, *DPYD*, *F5*, *G6PD*, *HLA-A*, *HLA-B* and *NUDT15*) showed that the vast majority of the Spanish population present a normal metaboliser status for these genes. Significantly, for some genes, more than 50% of the Spanish population tested showed a different metaboliser status than the normal one: for the *CYP3A5* gene, 87% of the population presented a poor metaboliser status and 12.4% an intermediate status; *VKORC1* and *CYP4F2* risk status was present in 65.3% and 56.3% of the population, respectively; as for the *CYP2C19* gene, 31% of the population present a rapid metaboliser status, 2% a poor metaboliser status, and 27.3% an intermediate status; for the *CYP2D6* gene, 4.5% of the population present a rapid metaboliser status, 2.2% a poor metaboliser status and 29.7% an intermediate status; and as for *IFNL3*, 52.5% present an unfavourable response phenotype. In the case of *DPYD*, although a high variability was detected, most of the variants were associated with normal function (c.1601G > A (*4), c.1672A > G (*5), c.1896T > C, c.2194G > A (*6), c.496A > G, c.85T > C (*9A)) not translating into a non-normal phenotype. Of note, other genes presented a moderate proportion of Spanish individuals with non-reference status, such as *CYP2B6* or *CYP2C9* (Figure 1B).

Finally, we found, based on the 21 PGx genes, that 98% of the Spanish population harbours at least one allele associated with a therapeutical change, with almost 60% of the population carrying more than 3 alleles in different genes, leading to changes in therapy (Figure 1C).

### 3.2. Therapeutical Impact of the Pharmacogenetic Landscape

**Figure 2:**
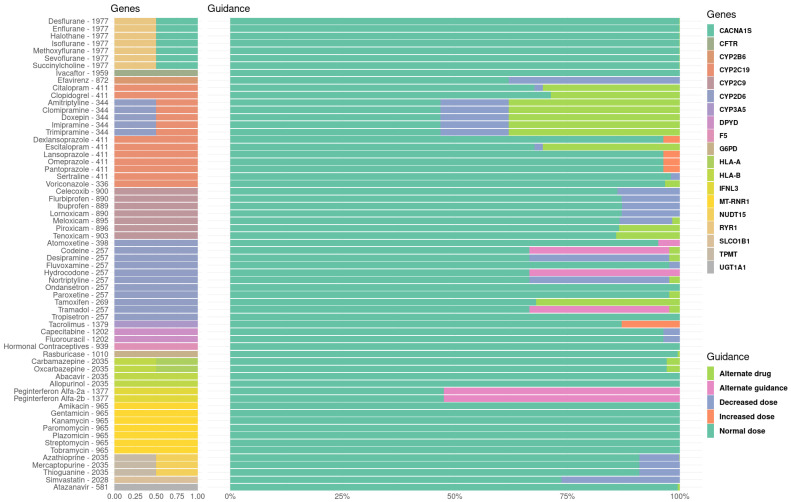
Therapeutical recommendations in the Spanish Population for drugs associated with PharmGKB level 1A genes. Drugs with available CPIC guidelines and associated with the 21 PGx genes categorised as level 1A in the PharmGKB database are included in the figure The number of individuals included for each drug is depicted next to the corresponding drug. The genes associated with the therapeutical prescription of the drugs are depicted in colours next to the corresponding drug. The proportion of the Spanish population is depicted with a normal dose (green), decreased dose (blue), increased dose (orange), alternate guidance (pink) and alternate drug (light green). No-call recommendations have been removed for visual purposes, but a full list of the recommendations identified is provided in Table S6.

A total of 64 drugs were obtained from the CPIC guidelines related with the 21 PGx genes evaluated. The transfer of the Spanish PGx characterisation to the clinical recommendation according to the CPIC guidelines is depicted in Figure 2 (Table S6). As expected, those drugs related with the more variable genes in our population showed the highest proportion of therapeutical changes. In this sense, those drugs associated with the genes *CYP2D6*, *CYP2C19*, *IFNL3* and *CYP2B6* are the most associated with a different therapeutic recommendation (Figure 2). In the case of *CYP3A5*, the predominance of *3 in Europe, with up to 85% of the individuals with a CYP3A5 non-expresser phenotype, has been widely described [41] and, in consequence, the standard starting dose is adjusted for non-expresser individuals, reducing the clinical impact of this high variability.

Interestingly, our study highlighted that patients prescribed with tricyclic antidepressants could benefit most from implementation, since around 50% of Spanish individuals would need the prescription of an alternative drug or a decrease in initial dose, as well as, for Peginterferon alfa-2a/2b. Around one-third of the Spanish individuals showed that they would need a different prescription for antiretroviral therapy (efavirenz), opioids and some chemotherapeutic agents (tamoxifen) (Figure 2). On the other hand, the NSAIDs family, simvastatin, anticoagulant therapy with clopidogrel and the immunosuppressor Tacrolimus seems to be altered in ~10–20% of the Spanish population. The remaining drugs seems to have less variability in the Spanish population, showing that a minor proportion of individuals would need a therapeutic change according to their PGx profile (Figure 2).

Regarding the clinical specialities, psychiatry—mainly through tricyclic antidepressants and selective serotonin reuptake inhibitors (SSRIs)-, infectious diseases (Peginterferon alfa-2a/2b and efavirenz) and pain management (opioids) were the clinical specialities with a greater proportion of the population needing a therapeutical intervention according to their PGx drug genotype, suggesting that these departments could benefit the most from the implementation of PGx testing.

Finally, we found that Spanish individuals would benefit from therapeutical change in a mean (min-max) of 3.31 drugs (0–25), considering the 64 drugs included in the CPIC guidelines and related with the 21 PGx genes evaluated.

### 3.3. Deleterious Variant Analysis

**Figure 3:**
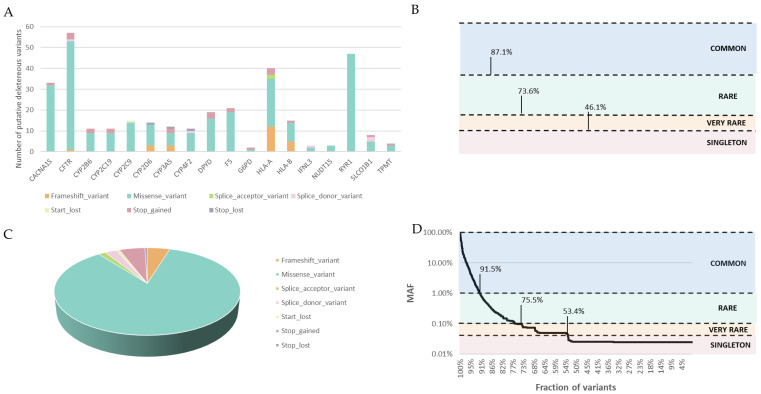
Putative deleterious variants identified in the 2046 individuals from exome and genome data. (**A**) Distribution of the putative deleterious variants identified in the 21 pharmacogenes categorised as level 1A in the PharmGKB database according to their consequence (Table S7). (**B**) Distribution of the variants identified in the 21 pharmacogenes categorised as level 1A in the PharmGKB database according to their MAF. Rare variants (MAF < 0.1%) and very rare variants (MAF < 0.01%) account for 87.1% and 73.6%, respectively, and 46.1% were identified only in one individual (singleton). (**C**) Distribution of the putative deleterious variants identified in the 1045 pharmacogenes included in the PharmGKB database according to their consequence (Table S8). (**D**) Distribution of the variants identified in the 1045 pharmacogenes included in the PharmGKB database according to their MAF. Rare variants (MAF < 0.1%) and very rare variants (MAF < 0.01%) account for 91.5% and 75.5%, respectively, and 53.4% were identified only in one individual (singleton).

Firstly, we identified 326 putative deleterious variants not related with PGx previously in 18 out of the 21 level 1A pharmacogenes evaluated. Putative deleterious variants were not identified in *VKORC1*, *UGT1A1* or *MT-RNR1*. According to their consequence type 82.5% were missense variants, followed by 7.4% frameshift, 6.7% stop gained, 1.5% splice donor, 0.9% stop lost, 0.6% splice acceptor and 0.3% start lost variants (Figure 3A, Table S7). The distribution of the variants through the genes showed a major proportion of putative deleterious variants in the genes *CFTR*, *RYR1*, *HLA-A* and *CACNA1S* (Figure 3). Interestingly, almost half of the missense variants were identified in three genes associated with low-frequency mutations (17.5% in *CFTR*, 14.4% in *RYR1*, and 10.1% in *CACNA1S*), whereas putative deleterious variants in CYPs genes were found in a lower proportion. Most of the putative deleterious variants were considered rare (87.1%, 0.1% < MAF < 1%), with 73.6% of the variants very rare (MAF < 0.1%) and, notably, 46.1% of the variants were detected in one individual only (Figure 3B). Despite the low frequency of the variants, 89.1% of the population harbours at least one putative deleterious variant in one actionable gene. Of note, 15 (4.6%) of the variants in 7 genes have not been described before in the gnomAD database, indicating that these variants could be private variants of the Spanish population (Table S7).

Secondly, with the aim of creating a catalogue of PGx putative deleterious variants in the Spanish population, we identified all the variants belonging to any of the 1045 genes described in PharmaGKB. We identified 7122 putative deleterious variants in 917 genes related with PGx (Table S8), with most of them being missense variants (84.9%), followed by stop gained variants (5.3%), frameshift (4.7%), splice donor (2.4%), splice acceptor (1.5%), start lost (0.7%) and stop lost variants (0.5%) (Figure 3C). We must note that most of the putative deleterious variants (90.6%) were identified in pharmGKB level 3 genes, level 1A (4.7%), level 2B (2.5%), level 2A (1.2%), level 4 (0.8%) and level 1B (0.2%). This finding correlates with the fact that more than 90% of genes contained in PharmGKB are categorised with a level 3 of evidence. As was found for 21 PGx genes, most of the putative deleterious variants were considered rare (91.5%), with 75.5% of the variants very rare and, notably, 53.4% of the variants were detected in one individual only (Figure 3B). It is noteworthy that 585 (8.21%) of the variants identified in 381 genes were not described before in the databases, indicating their possible private nature in the Spanish population.

### 3.4. Comparison of Different PGx Diagnostic Techniques

**Figure 4:**
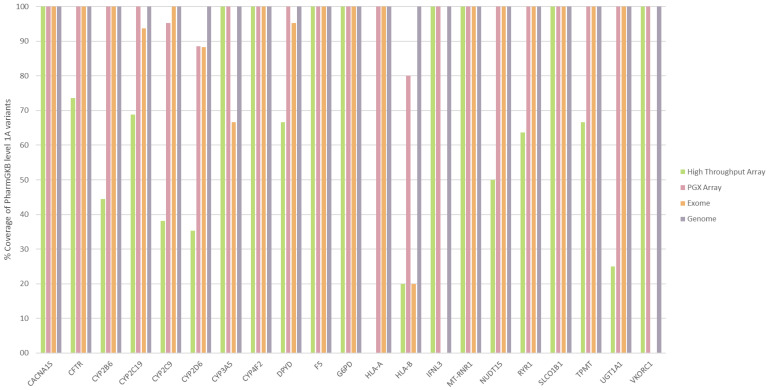
Coverage comparison of PharmGKB level 1A variants between different pharmacogenetic (PGx) diagnostic techniques. Percentage of coverage of the main PGx variants (PharmGKB level 1A variants) for the 21 main PGx genes (those associated with PharmGKB level 1A variants) is plotted for 4 PGx diagnostic techniques: standard high-throughput array (green), high-throughput array with enhanced PGx content (pink), whole-exome sequencing (orange) and whole-genome sequencing (purple).

The results from the comparison of the coverage of the 214 level 1A variants from the four techniques tested—(1) standard HT array, (2) PGx HT array, (3) WES and (4) WGS are depicted in Figure 4. As expected, WGS showed a coverage of 100% in all the genes evaluated, since, when enough coverage is used, the information from all genome positions is obtained with this technique. The second technique in terms of overall coverage of the level 1A variants was the PGx HT array (97.2%), followed by the WES (93.8%) and the standard HT array (58.3%).

Regarding the genes tested, those genes associated with a low number of variants, such as *CACNA1S*, *CYP4F2*, *F5*, *SLCO1B1* and *TPMT*, showed good coverage (mean coverage > 90%) of the main PGx variants in all the techniques (Figure 4). These genes are mainly associated with exonic variants, which are well covered by all techniques. Nevertheless, some genes contain clinically relevant variants (due to their frequency or their impact) located in non-exonic regions not allowing for the proper determination of the haplotype of these genes by means of exonic-focused techniques, such as WES (for example, CYP3A5*3 and CYP2C19*17, which are present in 92.4% [39] and 21.5% [40] of the Caucasian population, respectively). In addition, some genes associated exclusively with regulatory variants located in non-exonic regions (*IFL3* or *VKORC1*) or genetic complex regions (*HLA-B*) are not able to be determined by WES. In contrast, the coverage of the standard HT array is more related with the design of the array used. In this case, we have selected a HT array with an enhanced clinical content, but, even with this additional content, the coverage of the main variants is very limited (58.3%) and some genes, mainly CYPs genes, cannot be fully determined by this technique (Figure 4). On the contrary, a PGx HT array seems to be a good enough solution with overall coverage of 97.2% for the functional variants and covering all of the variants for 18 of the 21 genes analysed.

### 3.5. The Spanish Pharmacogenetic Database

**Figure 5:**
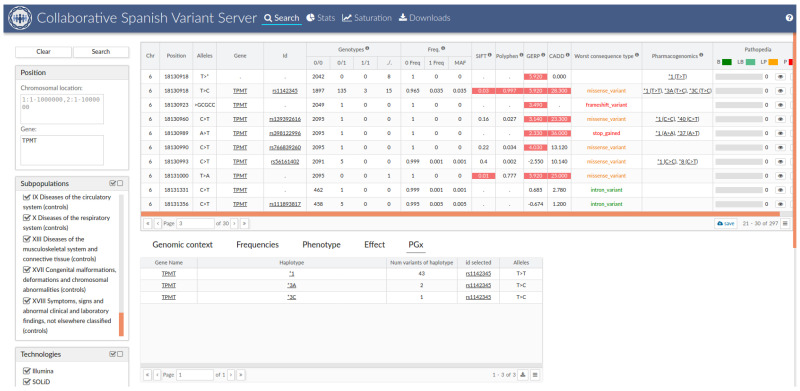
Screenshot of the Collaborative Spanish Variant Server (CSVS) v.5.0.0. showing an example of pharmacogenetic information of TPMT star (*) alleles or pharmacogenetic variants.

All the PGx information obtained in this study (single variant frequencies, *allele frequencies and putative deleterious variant frequencies) are available through the CSVS to provide information about the PGx variability of the Spanish population to the scientific/medical community [22] (Figure 5). This information is going to be updated with the new data uploaded to CSVS in order to provide a dynamic tool that contrasts with the static data usually available (i.e., on CPIC website).

## 4. Discussion

The implementation of PGx testing in clinical settings is one of the goals of current medicine to achieve safer and more effective treatments personalised for each patient. However, there are still some important points to facilitate the clinical implementation of PGx, and these points have been addressed in the current study. Firstly, our study provides information about reference PGx frequencies in the Spanish population by analysing the main 21 actionable PGx genes, thus being the most comprehensive study performed in such a population—with this information being provided on a gene server for the scientific community’s consultation. In this sense, our study provides the essential information to implement PGx testing in Spain in a directed way. Secondly, we have provided a catalogue of putative pathogenic variants with a potential impact in the drug response in 1045 genes previously related to PGx. Finally, we have undertaken comparison among the most commonly used HT PGx diagnostic techniques to offer information about which technique fits best for the different clinical settings. As a conclusion, our study provides valuable information about uncovered PGx testing aspects in order to help with the implementation of PGx in the clinical setting in Spain.

Although Spanish population has been described more diverse than Western and Northern Europeans [20], our PGx characterisation in the Spanish population showed similar frequencies to European populations [8,11] in the main 21 actionable PGx genes. This fact correlates with the evidence that the Spanish population is sufficient genetically similar to the Caucasian-European (CEU) population [20]. We only identified slight differences in some alleles of *CYP2B6*, *CYP2D6* and *HLA-A/B*, which could be explained by the differences in the *alleles’ calling tools, since most differences were found between *alleles with shared SNPs (i.e., CYP2B6*6/*9 or CYP2D6*1/*2). In the case of *HLA-A/B*, the differences could be mostly attributed to the sample size differences between our study’s population and the reference population, since the CPIC includes more than 1 million individuals for these specific alleles. Genes *CYP3A5*, *CYP2D6*, *VKORC1*, *CYP4F2* and *IFNL3* were identified as the most variable genes in the Spanish population, something which can be translated into a clear impact in terms of drug response. For instance, tacrolimus is the first-line immunosuppressant most widely prescribed in solid organ transplantation, with 40–50% of the variability in dose requirements for tacrolimus explained by SNVs in the *CYP3A5* gene, mainly the CYP3A5*3 allele [42]. Tacrolimus’s standard starting dose is adapted to the high prevalence of the CYP3A5 non-expresser genotype (homozygous for any of the non-functional alleles: *3, *6 or *7) in the Caucasian population and needs to be increased 1.5–2 times for CYP3A5 expresser phenotype individuals (homozygous for the reference allele or heterozygous for the previously mentioned non-functional alleles). Our study revealed that 13% of Spanish individuals will require an increased dose of tacrolimus to achieve successful immunosuppression, which is similar to the proportion reported for the Caucasian population [11].

The high degree of variability of *IFNL3*, *VKORC1* and *CYP4F2* identified leads to a high proportion of individuals who will need a therapeutical change of the associated drugs peginterferon-ribavirin and warfarin, respectively. Nevertheless, this fact does not translate into a clinical effect in the Spanish population since in Spain the use of peginterferon has been mostly replaced by direct-acting antiviral agents (DAAs), which achieve better sustained virologic responses in patients [43] and the use of warfarin is scarce [44]. On the contrary, acenocoumarol is the most used anticoagulant in the Spanish population [44] and, although there is no clinical guideline from the CPIC consortium, the DPWG group recommends a reduction of 50% of the initial dose in *VKORC1*-1639 (rs9923231) AA homozygous patients [9], which in our study represents 40% of the population, highlighting the potential impact in the Spanish clinical setting. Clopidogrel is also a standard antiplatelet therapy for patients with acute coronary syndrome, particularly in those undergoing percutaneous coronary intervention. Loss-of-function *CYP2C19* alleles have been associated with an increased risk of major adverse cardiovascular events [45]. Our study identified ~30% of the Spanish population with at least one non-functional allele and, therefore, these individuals would need an alternative therapy such as prasugrel or ticagrelor. As a result, anticoagulant therapies could have a significant benefit for inclusion in PGx testing in Spain.

Tricyclic antidepressants have also been identified to benefit from implementation in our country, since around 50% of Spanish individuals would need a therapeutical change. Despite the controversy about the use of PGx testing related with tricyclic antidepressants, there is substantial evidence supporting the effects of genetic variants in *CYP2D6*/*CYP2C9* in their metabolism [46]; thus, the high proportion of individuals whose genotype could affect these drugs’ metabolism points to the need to evaluate these drugs for prioritisation in terms of PGx implementation. In addition, selective serotonin reuptake inhibitors (SSRIs) associated with *CYP2D6*/*CYP2C19* also yielded around one-third of the individuals having an affected metabolism with these drugs, highlighting the importance of PGx testing in psychiatry and the great profit of implementing PGx in this clinical area [47]. Recently, opioids have been included in the new CPIC guidelines for the regulation of their PGx testing, mainly through *CYP2D6* determination [48]. Our study revealed that one-third of the Spanish population would need a different opioid prescription due to their *CYP2D6* genotypes. This high proportion of individuals (as well as the extensive use of opioids worldwide and, in particular, in Spain, where opioids are used by ~10% of the general population [49]) gives importance to the inclusion of opioids in the PGx testing strategy. Another drug mainly metabolised by CYP2D6 is the chemotherapeutic agent Tamoxifen. Despite the controversial results about the benefit of a *CYP2D6* guided treatment in Tamoxifen breast-cancer treated patients, the heterogeneous results seem to point to a benefit being yielded from *CYP2D6* determination in a homogeneous subset of patients [50]. As we have previously stated, one-third of our population showed abnormal metabolism of CYP2D6, requiring a therapeutical change to an alternative hormonal therapy in order to avoid a ~2- or 3-fold increased risk of breast cancer recurrence [51]. As a summary, due to the high degree variability identified in *CYP2D6*, which fully correlates with the previous description of the Caucasian population [11], drugs metabolised by this enzyme need to be prioritised for their PGx implementation in the clinical setting. In this sense, unlike previous studies [11], our study provides valuable data about the distribution of the CNVs in *CYP2D6*, thanks to the use of the PGx HT array. Therefore, we were able to determine that the contribution of CNVs in *CYP2D6* are scarce in our population due to the fact that most of the duplications are associated with the non-functional *4 allele. Nevertheless, testing CNVs in *CYP2D6* is essential to offer an accurate analysis of this gene.

Efavirenz is an antiretroviral used in HIV-infected patients which has a narrow therapeutic window between 1 and 4 μg/mL and with concentrations > 4 μg/mL, neurotoxicities such as ataxia or encephalopathy could appear [52]. Our population showed that ~40% of the individuals would need a decrease in the standard doses according to their *CYP2B6* genotype in order to avoid Central Nervous System side effects, as has also been described for the Caucasian population [11], pointing to the great importance of *CYP2B6* testing before Efavirenz prescription.

Simvastatin is a commonly used drug for cholesterol reduction worldwide. *SLCO1B1* genetic variants, in particular the rs4149056C allele, have been associated with higher risk of myopathy in simvastatin treated patients [53]. This decreased functional allele has been identified in 25% of Spanish individuals. Nevertheless, very recently a new update of the CPIC clinical guidelines has been published including new statins apart from simvastatin and new *SLCO1B1* decreased functional alleles, the rs2231142 variant in *ABCG2* and *CYP2C9* non-functional alleles [54]. These new genetic variants have not been explored in our study for simvastatin treatment, but since *CYP2C9* showed that ~35% of the population was carrying non-functional alleles, we could estimate than around ~50% of the population would need decreased doses of statins to successfully regulate cholesterol levels.

Some main anticancer drugs have been associated with germline PGx variants: tamoxifen (CYP2D6), thiopurines (*TPMT*, *NUDT15*) and fluoropyrimidines (*DPYD*). In contrast with the previously stated high proportion of individuals needing a therapeutical change for tamoxifen, the impact of the PGx variants in patients treated with thiopurines and fluoropyrimidines is lower, with 9% and ~4% of the individuals having an actionable genotype, respectively. Correlating with the previous frequencies reported for the Caucasian population [11,17], we found a higher contribution of *TPMT* genetic variants (8%), mainly TPMT*3A allele, than the *NUDT15* variants (<1%), which would entail a decrease in the thiopurines starting dose or an alternative drug in order to prevent a life-threatening myelosuppression [55]. In contrast, non-functional variants in the *DPYD* gene were associated with the develop of severe ADRs such as neutropenia, nausea, severe diarrhoea, or hand-foot syndrome in fluoropyrimidine-treated patients [56]. *DPYD* has been found to be highly polymorphic in our population, with almost half of the population carrying any variant, but when non-functional variants were considered, ~4% of the individuals would need a fluoropyrimidine decreased starting dose, since no *DPYD* poor metaboliser was identified. *DPYD* testing before starting fluoropyrimidine treatment has been recommended by international agencies for some years [57,58], but recently the European Medicines Agency has also included this recommendation [59] and, therefore, *DPYD* testing is increasingly present in the clinical setting in Europe. Although some of the anticancer drugs do not show a high proportion of individuals affected by a PGx variant, overall our study supports the evidence that around one-third of the patients had at least one actionable PGx variant which could impact the choice or dose of at least one anticancer drug [60], pointing to the great benefits of PGx germline testing in oncology services together with the widely implemented somatic genetic profiling in tumoral samples.

Finally, our study has provided information about the genetic frequencies of some genes not usually tested (*UGT1A1*, *G6PD*, *RYR1*-*CACNA1S*, *HLA-B* and *MT-RNR1*) due to the difficulty of determining the PGx alleles or their low coverage by the standard diagnostic techniques [11,17]. In this sense, our study provides reference frequencies of these genes to be used in the European population since no substantially differences has been observed among Spanish and other European populations. These genes showed no or very low variability and, therefore, their impact in the clinical setting in the Spanish population is very limited. This is the case of associated drugs: Aminoglycosides, Volatile Anaesthetic Agents, Atazanavir, Rasburicase, Abacavir and Allopurinol. Despite the severity of the ADRs in patients treated with these drugs, their extremely low prevalence in the Spanish population suggests that their implementation in our country could take second place, prioritizing other genes with a greater impact in our population, such as *CYP2D6*, *CYP2C19* or *CYP4F2*. In this study, we have transferred the PGx genotypes to the clinical impact in our population, which at the end is the final meaningful information needed to develop a PGx implementation strategy.

Genetic population differences in PGx genes leads to different ethnic sensitivities to drug response and, therefore, different proportions of pathological phenotypes. In this sense, rare variants have been described to contribute to inter-individual variability with a contribution from 1% of simvastatin metabolism to >40% of the contribution in the case of irinotecan [61]. Therefore, the identification of population-specific rare variants is essential for the better understanding of genomic effects on ethnic specific drug responses [62]. Our study provides a catalogue of putative pathogenic variants due to their consequence or their pathogenicity prediction not previously related with PGx. Regarding the 21 actionable PGx genes evaluated, although most of the variants identified are very rare (MAF < 0.01), we found that 89.1% of the population harbours at least one putative deleterious variant, highlighting the importance of the contribution of this kind of event in the PGx landscape. As expected, we found a large proportion of deleterious variants non-previously PGx related in those less studied genes, associated with low-frequency mutations (*CFTR*, *RYR1*, *CACNA1S*) as has been reported in other populations [17,18]. This fact supports the previous evidence that contributions of rare genetic variants are gene and drug specific [61]. Therefore, these genes with a major contribution of rare deleterious variants could be good candidates to be implemented using NGS technologies which allow for the detection of such events. Finally, although some concerns exist regarding the deleteriousness predictors specifically in PGx genes due to their poor conservation [63], we have used solid predictors for the evaluation of the deleteriousness of the variants, especially in missense variants where at least two predictors have to coincide in order to be classified as putative deleterious. However, the aim of our study is to provide a catalogue of the putative deleterious variants available to the scientific community so that they can be functionally evaluated before their inclusion in clinical guidelines.

PGx diagnostic has been demonstrated to be cost-effective and even cost-saving in most of the drugs with associated clinical guidelines [63]. In this sense, to assure the cost-effectiveness, the election of the molecular technique to assess the PGx genotypes is key. Nowadays, the range and types of techniques available to perform PGx diagnostics are very wide, from single variant testing (Sanger sequencing or allelic discrimination) to massive testing for pre-emptive PGx diagnostics. Although HT-techniques are more expensive they are more informative evaluating hundreds of PGx markers at once. In this last case, there are some options available such as HT genotyping with SNP arrays, WES and WGS. All these techniques have different positive and negative aspects, which need to be considered before their choice for PGx implementation. In this study, we have evaluated and compared the ability of these techniques to cover all the actionable variants in the 21 PGx genes leading to a therapeutic change. Our comparative analysis shows, as expected, that WGS offers the best coverage of the variants allowing for not only detection of all SNVs but also, for actionable CNVs such as *CYP2D6*, complete deletion or duplication. Nevertheless, although WGS would be the preferable PGx diagnostic technique as has been described in some PGx studies [16], the cost of this technique is higher and, therefore, its implementation in the clinical setting is harder [64]. On the contrary, the PGx HT array shows a coverage of actionable SNVs of 97.6%, allowing us to also determine *CYP2D6* CNVs with a very low cost. Nevertheless, since WES is becoming widely used in the clinical setting (for example for rare disease diagnosis), several studies have employed this technique in order to perform a PGx characterisation [11,15,17]. All these studies are focused only on the subset of genes that are well covered for this technique, correlating with our study that shows an overall coverage with WES of 93.8% of all the actionable variants, and the lack of capacity to analyse the relevant genes with variants in intronic or promoter regions such as *CYP2C19*, *CYP3A5*, or *IFNL3*, as well as *CYP2D6* CNVs. It must be noted that some of these not fully covered genes present high variability in some populations, such as *CYP2C19* and *CYP3A5*, with 20% and 92.6% of the Spanish population carrying *alleles not covered by WES. In this sense, the use of WES as a choice technique for PGx’s pre-emptive diagnostic is limited by its coverage but also by its cost and, therefore, a combination with an additional technique would be desirable. Finally, standard HT arrays show the lowest coverage of all actionable variants (58.3%) and, although genetic imputation could be performed, the genomic imputation showed unequal accuracy between PGx genes and populations [11], suggesting that these arrays are not the best option for PGx pre-emptive diagnostics. As a conclusion, WGS would be the best choice for PGx pre-emptive diagnostics, but although its cost is becoming cheaper, it is still too high to be implemented in the clinical practice. In this sense, according to our data, the use of a PGx HT array seems to be the best alternative. With a reasonable cost, easy-to-interpret data and no computer analysis costs requirement, this type of arrays cover an enormous number of PGx markers including CNVs.

One of the main strengths of this study is the creation of a PGx database to integrate all the data generated in our analysis. Unlike the global databases (i.e., gnomAD [38], Hapmap [65], 1000G project [24]) which have a dynamic structure with automatic updates when new data are uploaded, reference PGx data are provided in a static and difficult-to-manage way (i.e., the CPIC guidelines [8] provide static excel or PDF files to be explored as supplementary files of the guidelines themselves). In the new, big data analysis era, it becomes necessary for PGx data tools to be accessed in an automated way in order to compare the results through computing tools. Our PGx-CSVS database provides a constantly updated database which is able to perform PGx interpretation for the new data uploaded and allows access to all this information in an automated way. To our knowledge, this is the first fully updated PGx database created to the date.

This study has some limitations, since the data used for the study is non-homogeneous data obtained from different techniques (HT arrays, WES and WGS) with specific genomic design and coverage. To ensure the reliability of the PGx genotypes, we have included only the data of those individuals with all the essential variants covered for each gene. Consequently, characterisation of all the actionable genes is not covered by the same number of individuals. However, due the great number of individuals included in our study and the frequency of the most relevant PGx variants, the frequency data provided in this study are informative enough to be used as a reference for the population. Nevertheless, this limitation is also a strength in our study, since we circumvent other limitations based on technique design (such as WES studies, which allow only for the characterisation of a subset of genes) and, in addition, we have been able to perform a PGx technique comparison that yields valuable information about the different techniques. Lastly, in our study we have only included individuals born in Spain to evaluate the genetic variability in the Spanish population. Nevertheless, 11.6% of Spanish population in 2022 were immigrant according to the Spanish National Statistical Institute, and it is necessary to consider this fact, since other genetic ancestries could be found in the clinical reality.

In conclusion, our study provides valuable PGx reference information on the Spanish population to help with the implementation of PGx diagnostics in this region, but more importantly it offers an updated tool to be used as a reference in the Spanish and European population. Additionally, this study provides relevant information to help with the implementation of PGx diagnostics worldwide, tackling some of the main issues of PGx implementation.

## Data Availability

All the pharmacogenetic data generated in this study is available at Collaborative Spanish Variant Server to be explored and downloaded: http://csvs.babelomics.org/ (accessed on 15 October 2021).

## Supplementary Materials

The following supporting information can be downloaded at: https://www.mdpi.com/article/10.3390/pharmaceutics15041286/s1, Table S1: The 21 actionable pharmacogenes, categorised as 1A by PharmGKB database and their genomic positions. A total of 5 kb upstream and downstream surrounding the gene were included in the analysis to obtain the pharmacogenetic variants. Genomic positions are described in GRCh37/hg19 genome version. Table S2: All genes included in PharmGKB database (1045 genes) and PharmGKB evidence level. Table S3: Pharmacogenetic alleles and variants for 21 main pharmacogenetic genes identified in Spanish population. Table S4: Pharmacogenetic diplotypes for 21 main pharmacogenetic genes identified in Spanish population. Comparisons between diplotype frequencies (without not-called data) obtained and CPIC’s frequencies in European population (EUR) have been performed with Fisher’s exact test. P-value and odd ratio are depicted in the table. Table S5: Pharmacogenetic phenotypes for 21 main pharmacogenetic genes identified in Spanish population. Table S6: Pharmacogenetic phenotypes for 21 main pharmacogenetic genes identified in Spanish population. Table S7: Putative deleterious variants identified in actionable genes, categorised as 1A by PharmGKB database. Table S8: Putative deleterious variants identified in the 1045 genes included PharmGKB database. Figure S1: Summary diagram of the Study. Main objectives of the study are represented.
