# Supplementary figures and images for: A Comprehensive Analysis of 21 Actionable Pharmacogenes in the Spanish Population: From Genetic Characterisation to Clinical Impact
Source: Pharmaceutics. 2023 Apr 19;15(4):1286. doi: 10.3390/pharmaceutics15041286 (PMC10140932; doi:10.3390/pharmaceutics15041286)

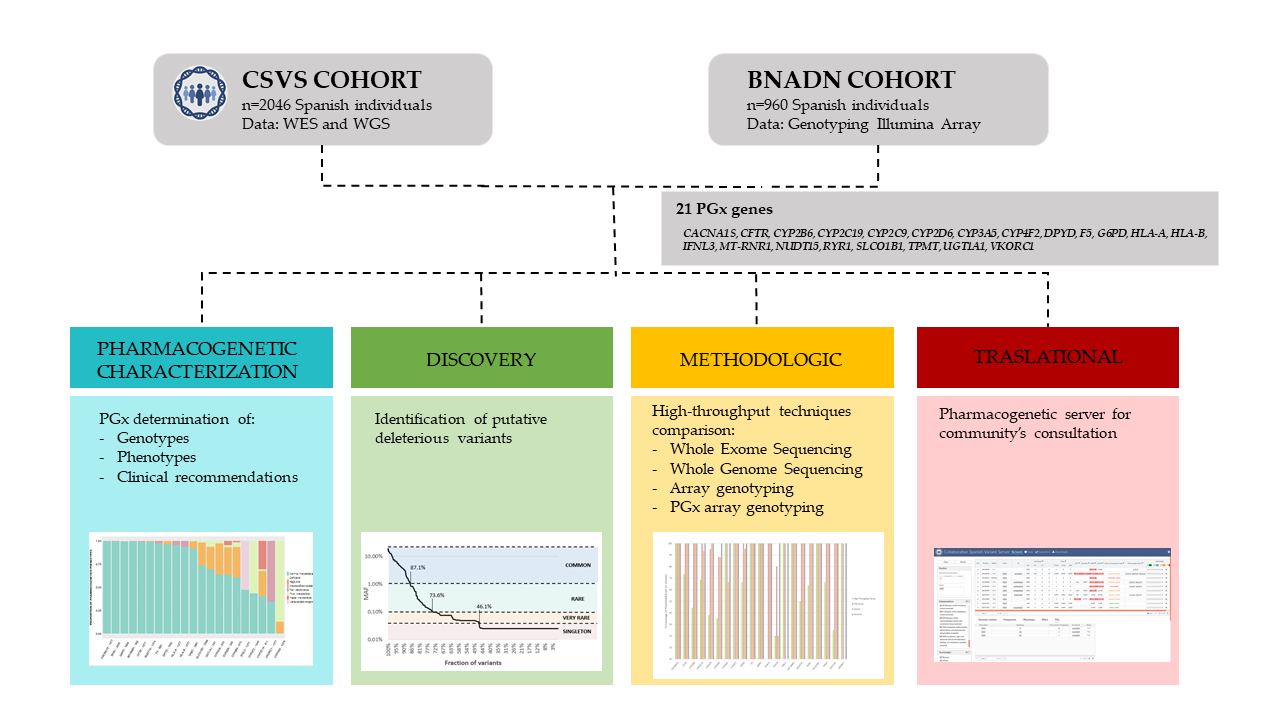


**Figure S1.** Summary diagram of the Study. Main objectives of the study are represented.

Supplement: Supplementary file 1 [file pharmaceutics-15-01286-s001.zip › pharmaceutics-2305883-Figure S1.docx]
